# Supplementary material for: Are There Ethnic Differences in Recorded Features among Patients Subsequently Diagnosed with Cancer? An English Longitudinal Data-Linked Study
Source: Cancers (Basel). 2023 Jun 7;15(12):3100. doi: 10.3390/cancers15123100 (PMC10296232; doi:10.3390/cancers15123100)
Supplement: Supplementary file 1 [file cancers-15-03100-s001.zip › cancers-2408136-supplementary.pdf]

Supplementary File S1: Analysis of patients without relevant recorded features

We described demographics and percentage by ethnicity of patients without relevant recorded features (Supplementary file S3a and S3b below). Logistic regression models examined the association between ethnicity and having any relevant recorded feature before diagnosis. Analysis was adjusted for age, sex, comorbidity scores, Index of Multiple Deprivation, and smoking status.

Supplementary File S2: Results of analysis of patients without features by ethnicity

Demographically, participants with or without relevant features were broadly similar, except for sex, co-existing conditions, and cancer types (Supplementary File 3a and 3b above). Those without features had a lower percentage with comorbidities than those with features (83% vs 93%). Breast (26%) and colorectal (24%) cancer were the most common among those without features, whereas prostate (30%) and lung (25%) were more common among those with features. Reflecting the differences in cancer sites, around half of the participants without features were females: ranging from 39% in the Black group to 53% in the Asian and Mixed groups. Three-fifths of those with features were males: ranging from 57% among Asians to 71% in the Black group. Compared with the White patients (and accounting for cancer and demographic factors), Black [Adjusted Odds Ratio (AOR)=0.86, 95% CI:0.80-0.91, $p<0.0001$] and Asian patients (AOR=0.84, 95%CI:0.78-0.89, $p<0.0001$) were somewhat less likely to have recorded features, with the opposite pattern seen in Mixed ethnicity patients (AOR=1.20, 95%CI:1.12-1.29, $p<0.0001$).

Supplementary Table S1: Characteristics of participants without relevant recorded features by ethnicity

		White	Black	Asian	Mixed	Other	All
**Age (years)**	Median (IQR)	72 (62-81)	64 (53-75)	65 (54-75)	71 (60-79)	72 (62-82)	71 (61-81)
**Sex**	Male n (%)	33,070 (49.1)	1,044 (61.1)	677 (47.5)	513 (47.4)	1,012 (49.9)	36,316 (49.4)
**IMD n (%) ***	*1 (least deprived)*	15,638 (23.3)	71 (4.16)	237 (16.3)	178 (16.5)	513 (25.3)	16,637 (22.6)
	2	14,389 (21.4)	106 (6.21)	228 (16.0)	170 (15.7)	459 (22.6)	15,352 (20.9)
	3	13,359 (19.9)	282 (16.5)	277 (19.5)	213 (19.7)	406 (20.0)	14,537 (19.8)
	4	12,414 (18.5)	496 (29.1)	360 (25.3)	258 (23.8)	347 (17.1)	13,875 (18.9)
	5 (most deprived)	11,443 (17.0)	752 (44.1)	322 (22.6)	263 (24.3)	305 (15.0)	13,085 (17.8)
Morbidity score* n (%)	*0 - None*	11,207 (16.7)	407 (23.8)	322 (22.6)	132 (12.2)	532 (26.2)	12,600 (17.1)
	1	14,855 (22.1)	400 (23.4)	289 (20.3)	201 (18.6)	524 (25.8)	16,269 (22.1)
	2	13,988 (20.8)	353 (20.7)	335 (23.5)	198 (18.3)	394 (19.4)	15,268 (20.8)
	3	13,934 (20.7)	274 (16.0)	236 (16.6)	266 (24.6)	344 (16.9)	15,054 (20.5)
	4 (most score)	13,307 (19.8)	274 (16.0)	243 (17.1)	285 (26.3)	236 (11.6)	14,345 (19.5)
Smoking status, n (%)	*Current smoker*	14,773 (21.9)	367 (21.5)	183 (12.8)	247 (22.8)	525 (25.9)	16,095 (21.9)
	Never smoked	23,750 (35.3)	846 (49.5)	875 (61.4)	399 (36.9)	751 (37.0)	26,621 (36.2)
	Ex-smoker	21,315 (31.7)	302 (17.7)	191 (13.4)	310 (28.7)	520 (25.6)	22,638 (30.8)
	Unknown	7,453 (11.1)	193 (11.3)	176 (12.4)	126 (11.7)	234 (11.5)	8,182 (11.1)
Sites, n (%)	Breast	17,351 (25.8)	392 (22.9)	479 (33.6)	317 (29.3)	445 (21.9)	18,984 (25.8)
	Lung	14,937 (22.2)	216 (12.7)	234 (16.4)	192 (17.7)	498 (24.5)	16,077 (21.9)
	Prostate	10,833 (16.1)	587 (34.4)	227 (15.9)	176 (16.3)	273 (13.5)	12,096 (16.5)
	Colorectal	16,451 (24.5)	268 (15.7)	301 (21.1)	270 (24.9)	544 (26.8)	17,834 (24.3)
	Oesophagogastric	5,588 (8.30)	110 (6.44)	106 (7.44)	92 (8.50)	216 (10.6)	6,112 (8.31)
	Myeloma	2,131 (3.17)	135 (7.90)	78 (5.47)	35 (3.23)	54 (2.66)	2,433 (3.31)
	**Total**	**67,291 (91.5)**	**1,708 (2.32)**	**1,425 (1.94)**	**1,082 (1.47)**	**2,030 (2.76)**	**73,536 (100)**

Supplementary Table S2: Percentage with relevant recorded features by ethnicity

Sites	Feature	All (N=188,487)		White (N=174,171)		Black (N=3,477)		Asian (N=2,945)		Mixed (N=3,493)		Other (N=4,392)	
		Total n (%)	Percent recorded as isolated	Total n (%)	Percent recorded as isolated	Total n (%)	Percent recorded as isolated	Total n (%)	Percent recorded as isolated	Total n (%)	Percent recorded as isolated	Total n (%)	Percent recorded as isolated
Breast	Breast pain	1,116 (5.24)	75.5	995 (5.10)	76.3	33 (8.13)	54.6	39 (6.90)	58.9	28 (6.24)	89.3	21 (5.36)	85.7
	Breast lump	19,563 (91.8)	98.1	17,915 (91.9)	98.1	362 (89.2)	95.0	517 (91.5)	96.9	410 (91.3)	99.0	359 (91.6)	98.3
	Breast skin changes	143 (0.67)	62.9	135 (0.69)	65.2	2 (0.49)	0.0	1 (0.18)	50.0	1 (0.22)	0.0	3 (0.77)	33.3
	Nipple discharge	353 (1.66)	73.4	323 (1.66)	73.1	9 (2.22)	66.7	6 (1.06)	83.3	8 (1.78)	75.0	7 (1.79)	85.7
	Nipple Retraction	95 (0.45)	71.6	92 (0.47)	71.7	0 (0)	0.0	0 (0)	0.0	2 (0.45)	50.0	1 (0.26)	100
	Lymphadenopathy (axilla)	35 (0.16)	65.7	33 (0.17)	66.7	0 (0)	0.0	1 (0.18)	100	0 (0)	0.0	1 (0.26)	0.0
	Total	**21,305**	96.1	**19,493**	96.2	**406**	90.6	**565**	93.9	**449**	97.6	**392**	96.7
Lung	Appetite loss	598 (0.98)	15.4	552 (0.97)	15.4	7 (1.39)	71.4	8 (1.11)	12.5	9 (0.86)	0.0	22 (1.24)	4.55
	Chest infection	8,818 (14.4)	21.5	8,280(14.5)	21.5	57 (11.3)	22.8	92 (12.8)	20.7	140 (13.4)	15.7	249 (14.0)	23.3
	Chest pain	4,613 (7.54)	22.7	4,303 (7.53)	22.6	38 (7.54)	26.3	66 (9.18)	33.3	74 (7.08)	22.9	132 (7.42)	19.7
	Chest signs	1,531 (2.50)	16.0	1,420 (2.48)	16.1	14 (2.78)	21.4	23 (3.20)	13.0	24 (2.30)	16.7	50 (2.81)	14.0
	Cough	11,579 (18.9)	24.5	10,802 (18.9)	24.5	113 (22.4)	33.6	178 (24.8)	29.2	180 (17.2)	19.4	306 (17.2)	22.6
	Dyspnoea	12,085 (19.8)	26.5	11,308 (19.8)	26.4	85 (16.9)	34.1	107 (14.9)	19.6	262 (25.1)	30.5	323 (18.2)	25.4
	Fatigue	1,894 (3.10)	17.4	1,782 (3.12)	17.7	15 (2.98)	26.7	20 (2.78)	15.0	31 (2.97)	6.45	46 (2.59)	10.9
	Lung metastases	1,608 (2.63)	25.4	1,505 (2.63)	25.1	15 (2.98)	40.0	16 (2.23)	12.5	20 (1.91)	30.0	52 (2.92)	30.8
	Finger clubbing	126 (0.21)	22.2	110 (0.19)	20.9	3 (0.60)	0.0	4 (0.56)	50.0	1 (0.10)	0.0	8 (0.45)	37.5
	Haemoptysis	2,315 (3.78)	25.2	2,172 (3.80)	24.7	27 (5.36)	48.2	37 (5.15)	21.6	30 (2.87)	26.7	49 (2.76)	36.7
	Hoarseness	636 (1.04)	23.4	595 (1.04)	22.9	8 (1.59)	50.0	8 (1.11)	37.5	9 (0.86)	0.0	16 (0.90)	37.5
	Lymphadenopathy	330 (0.54)	23.6	308 (0.54)	24.0	2 (0.40)	50.0	6 (0.83)	0	2 (0.19)	0.0	12 (0.67)	25.0
	Shoulder pain	1,794 (2.93)	20.8	1,664 (2.91)	20.4	22 (4.37)	36.4	28 (3.89)	17.9	31 (2.97)	25.8	49 (2.76)	24.5
	Signs of SVC obstruction	147 (0.24)	19.7	141 (0.25)	18.4	1 (0.20)	0.0	0.0	0.0	2 (0.19)	0.0	3 (0.17)	100
	Stridor	32 (0.05)	6.25	28 (0.05)	7.14	1 (0.20)	0.0	0.0	0.0	1 (1.10)	0.0	2 (0.11)	0.0
	Thrombocytosis	6,672 (10.9)	24.7	6,198 (10.9)	24.7	40 (7.94)	27.5	57 (7.93)	15.8	106 (10.1)	15.1	271 (15.2)	29.9
	Weight loss	2,018 (3.30)	21.1	1,872 (3.28)	21.2	17 (3.37)	23.5	19 (2.64)	21.1	44 (4.21)	11.4	66 (3.71)	22.7

	X-ray findings	4,396 (7.18)	17.8	4,106 (7.19)	18.1	39 (7.74)	15.4	50 (6.95)	8.00	79 (7.56)	7.59	122 (6.86)	20.5
	**Total**	**61,192**	23.1	**57,146**	23.1	**504**	30.8	**719**	21.9	**1,045**	20.0	**1,778**	24.2
	Abnormal DRE	752 (1.45)	5.05	683 (1.43)	5.12	19 (1.16)	5.26	19 (2.41)	5.26	12 (1.08)	0.0	19 (2.21)	5.26
	Erectile dysfunction	2,094 (4.02)	12.2	1,844 (3.87)	11.7	130 (7.93)	15.4	47 (5.96)	19.2	53 (4.76)	20.8	20 (2.33)	5.00
**Prostate**	Haematuria, visible	3,125 (6.01)	29.9	2,917 (6.12)	29.8	65 (3.96)	30.8	43 (5.46)	30.2	57 (5.12)	28.1	43 (5.00)	37.2
	LUTS	10,168 (19.5)	16.8	9,375 (19.7)	16.9	286 (17.4)	17.5	148 (18.8)	13.5	202 (18.1)	13.4	157 (18.3)	16.6
	Raised PSA	35,900 (68.9)	68.8	32,818 (68.9)	68.7	1,140 (69.5)	68.9	531 (67.4)	67.0	790 (70.9)	71.4	621 (72.2)	73.6
	Total	**52,039**	53.1	**47,637**	53.0	**1,640**	53.4	**788**	50.6	**1,114**	55.5	**860**	58.3
	Abdominal mass	481 (1.66)	29.1	444 (1.64)	28.8	8 (1.94)	37.5	4 (0.88)	25.0	9 (1.80)	22.2	16 (2.47)	37.5
	Abdominal pain	6,979 (24.0)	62.1	6,467 (23.9)	62.3	107 (25.9)	58.8	119 (26.2)	54.6	116 (23.2)	58.6	170 (26.3)	64.1
	Change in bowel habit	3,269 (11.3)	67.6	3,109 (11.5)	67.7	28 (6.80)	57.1	27 (5.93)	55.6	38 (7.58)	73.7	67 (10.4)	67.2
	Faecal occult blood	174 (0.60)	40.2	162 (0.60)	40.1	3 (0.73)	33.3	2 (0.44)	50.0	5 (1.00)	40.0	2 (0.31)	50.0
**Colorectal**	Iron-deficiency anaemia	10,702 (36.9)	68.1	9,945 (36.8)	68.3	156 (37.9)	57.7	181 (39.8)	62.4	191 (38.1)	70.2	229 (35.4)	68.6
	Rectal bleeding	6,094 (20.9)	72.9	5,662 (20.9)	73.2	86 (20.9)	69.8	108 (23.7)	67.6	119 (23.8)	73.1	119 (18.4)	71.4
	Rectal mass	159 (0.55)	52.2	151 (0.56)	51.7	3 (0.73)	66.7	0 (0)	0.0	1 (0.20)	100.0	4 (0.62)	50.0
	Weight loss	1,175 (4.05)	36.9	1,078 (3.99)	37.0	21 (5.10)	9.52	14 (3.08)	35.7	22 (4.39)	27.3	40 (6.18)	52.5
	**Total**	**29,033**	65.5	**27,018**	65.7	**412**	57.5	**455**	60.0	**501**	65.5	**647**	65.8
	Back pain	1,181 (6.17)	22.8	1,091 (6.17)	22.6	20 (6.87)	20.0	23 (8.33)	17.4	18 (6.21)	27.8	29 (4.82)	34.5
	Dyspepsia	2,817 (14.7)	32.5	2,621 (14.8)	32.6	33 (11.3)	30.3	31 (11.2)	12.9	42 (14.5)	35.7	90 (14.9)	36.7
	Dysphagia	3,871 (20.2)	59.0	3,622 (20.5)	59.2	31 (10.7)	58.1	54 (19.6)	53.7	52 (17.9)	57.7	112 (18.6)	57.1
	Haematemesis	341 (1.78)	2.64	316 (1.79)	2.53	10 (3.44)	10.0	4 (1.45)	0.0	4 (1.38)	0.0	7 (1.16)	0.0
	Low haemoglobin*	2,705 (14.1)	32.7	2,459 (13.9)	32.7	65 (22.3)	43.1	52 (18.8)	21.2	47 (16.2)	36.2	82 (13.6)	29.3
**Oesophago-gastric**	Nausea	463 (2.42)	17.9	441 (2.49)	18.8	5 (1.72)	0.0	3 (1.09)	0.0	5 (1.72)	0.0	9 (1.50)	0.0
	Reflux	1,663 (8.69)	19.2	1,553 (8.79)	19.5	19 (6.53)	21.1	21 (7.61)	14.3	23 (7.93)	8.70	47 (7.81)	17.0
	Suspicious barium meal	12 (0.06)	41.7	11 (0.06)	36.4	1 (0.34)	100	0	0.0	0	0.0	0	0.0
	Thrombocytosis	1,918 (10.0)	19.1	1,758 (9.95)	19.3	25 (8.59)	12.0	23 (8.33)	21.7	34 (11.7)	14.7	78 (12.9)	17.9
	Upper abdominal mass	16 (0.08)	37.5	11 (0.06)	27.3	1 (0.34)	100.0	0	0.0	2 (0.69)	0.0	2 (0.33)	100.0
	Upper abdominal pain	2,111 (11.0)	38.4	1,912 (10.8)	38.6	46 (15.8)	28.3	47 (17.0)	46.8	36 (12.4)	36.1	70 (11.6)	35.7

	vomiting	1,107 (5.78)	21.7	1,020 (5.77)	21.9	18 (6.19)	22.2	14 (5.07)	0.0	11 (3.79)	9.09	44 (7.31)	27.3
	Weight loss	931 (4.87)	33.6	862 (4.88)	33.4	17 (5.84)	58.8	4 (1.45)	0.0	16 (5.52)	25.0	32 (5.32)	34.4
	Total	**19,136**	34.0	**17,677**	34.2	**291**	33.3	**276**	28.3	**290**	31.7	**602**	33.7
**Myeloma**	Bone pain	102 (1.76)	40.2	95 (1.83)	41.1	3 (1.34)	0.0	1 (0.66)	0.0	2 (2.13)	50.0	1 (0.88)	100
	Back pain	1,375 (23.8)	39.8	1,234 (23.7)	39.1	43 (19.2)	51.2	40 (26.5)	40.0	26 (27.7)	57.7	32 (28.3)	34.4
	Bence-Jones protein	666. (11.5)	21.8	600 (11.5)	22.2	29 (12.9)	17.2	16 (10.6)	6.25	8 (8.51)	50.0	13 (11.5)	15.4
	Abnormal ESR	1,370 (23.7)	29.4	1,216 (23.4)	29.4	60 (26.8)	33.3	49 (32.5)	26.5	22 (23.4)	27.3	23 (20.4)	26.1
	Hypercalcaemia	463 (8.01)	19.2	421 (8.10)	19.2	15 (6.70)	26.7	7 (4.64)	0.0	11 (11.7)	36.4	9 (7.96)	0.0
	Abnormal WBC	1,000 (17.3)	34.0	889 (17.1)	33.8	54 (24.1)	44.4	24 (15.9)	29.2	14 (14.9)	35.7	19 (16.8)	21.1
	Pathological fracture	23 (0.40)	73.9	21 (0.40)	76.2	1 (0.45)	0.0	1 (0.66)	100	0 (0)	0.0	0 (0)	0.0
	Paraprotein	414 (7.16)	15.2	374 (7.19)	14.7	14 (6.25)	21.4	11 (7.28)	18.2	6 (6.38)	16.7	9 (7.96)	22.2
	Plasma viscosity	351 (6.07)	30.1	333 (6.40)	31.2	5 (2.23)	40.0	2 (1.32)	0.0	5 (5.32)	0.0	6 (5.31)	0.0
	Spinal cord compression	18 (0.31)	44.4	17 (0.33)	41.2	0 (0)	0.0	0 (0)	0.0	0 (0)	0.0	1 (0.88)	100
	Total	**5,782**	30.4	**5,200**	30.3	**224**	35.7	**151**	26.5	**94**	38.3	**113**	23.9

*SVC: superior vena cava, DRE: digital rectal examination, PSA: prostate-specific antigen, * low haemoglobin with gastrointestinal bleeding, ESR: erythrocyte sedimentation rate, WBC: white blood cell count.*

Supplementary Figure S1: Flowchart of exclusion process

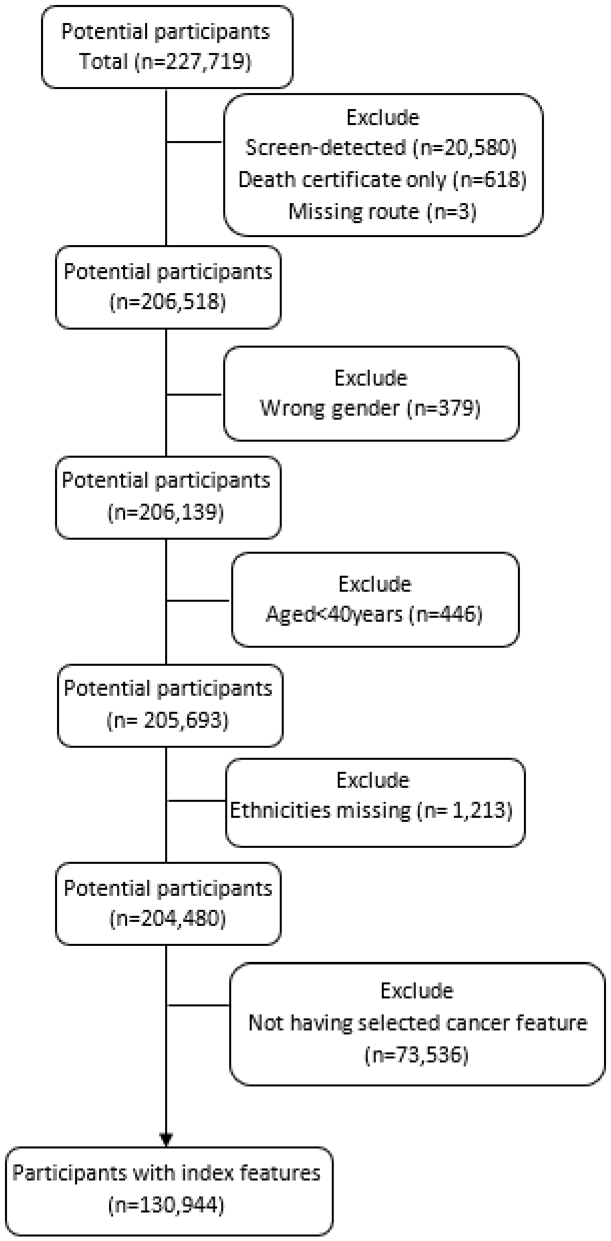

Supplementary Figure S2: Percentage without relevant recorded features, by ethnicity

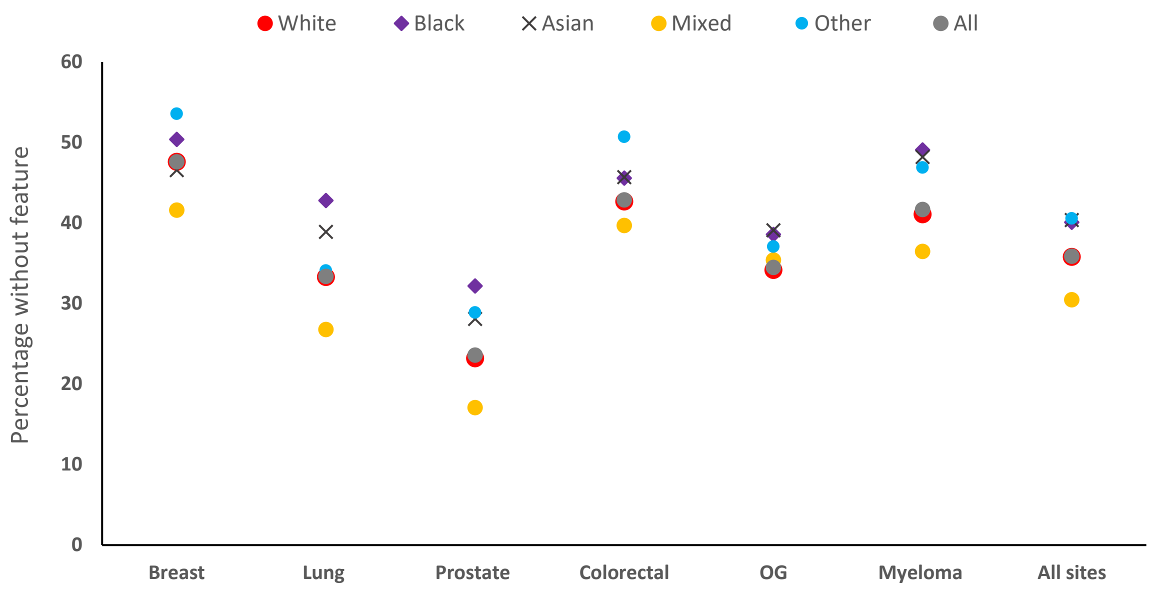

OG: Oesophagogastric. The total number without recorded features each site: [breast (n=18,984), lung (n=16,077), prostate (n=12,096), colorectal (n=17,834), oesophagogastric (n=6,112), myeloma (n=2,433), and All sites (n=73,536)]. Patients without recorded features: White (n=67,291), Black (n=1,708), Asian (n=1,425), Mixed (n=1,082), and Other (n=2,030)
